# From family trials to genomic mate allocation: statistical and genomic strategies to accelerate sugarcane genetic improvement

**DOI:** 10.1007/s00122-026-05373-9

**Published:** 2026-09-12

**Authors:** Andrew Rigby, Felicity Atkin, Ben Hayes, Lee Hickey, Seema Yadav

**Affiliations:** 1 Sugar Research Australia, Meringa, Gordonvale, QLD Australia; 2 PT. Global Papua Abadi, Sermayam, Indonesia; 3 Queensland Alliance for Agriculture and Food Innovation, Queensland Bioscience Precinct, St. Lucia, Brisbane, QLD Australia

## Abstract

Sugarcane (*Saccharum* spp.) underpins global sugar and bioenergy supply and is increasingly valued as a renewable biomass feedstock. Sustained improvement in commercial traits and resilience is constrained by long breeding cycles, clonal propagation, multi-stage testing, and a highly polyploid, heterozygous, and frequently aneuploid genome with substantial non-additive genetic variation. Genomic selection has demonstrated value for predicting elite-clone performance, yet its operational use remains limited at earlier decision points, including family selection, parent evaluation, and cross design. This review examines the biological, statistical, and genomic factors that shape these decisions, with emphasis on the Australian breeding context based on progeny assessment trials (PATs), clonal assessment trials (CATs), and final assessment trials (FATs). We evaluate challenges arising from family plot means, the use of different full-sib samples as nominal family replicates, spatial heterogeneity, competition, genotype-by-environment interaction, and the partitioning of additive and non-additive effects. We also assess the integration of pedigree and genomic relationship, genotype representation, allele-dosage estimation, aneuploidy, genomic prediction models, and training-population design. We then consider genomic prediction of cross performance and constrained mate allocation as approaches for improving expected family performance, accounting for cross-specific non-additive effects and managing relatedness. We propose a decision-centred framework that links family and clonal data across breeding stages, tracks the propagation of information and uncertainty, and supports parent recycling and cross allocation. We conclude with a practical research agenda for stage-integrated mixed-model and single-step analyses that connect early family evaluation with genomic prediction and cross-level decision support in sugarcane breeding.

## Introduction

Global agriculture is being reshaped by rising demand, climate volatility, tightening resource constraints, and increasing input costs (Mihrete and Mihretu 2025). In this context, genetic improvement remains one of the most durable levers for lifting productivity and resilience while reducing the resource footprint of production systems. Sugarcane is a uniquely strategic crop because it simultaneously supports food, materials, and energy markets through sucrose, fibre, and high biomass output (Alonso-Pippo et al. 2013; Kahn and Kahn 2019). Its capacity for high dry matter production and adaptability across diverse environments position it as a key contributor to bioenergy and bioproduct supply chains, alongside its central role in sugar markets (Santos et al. 2020; Mod et al. 2024).

Despite steady progress from conventional breeding, sugarcane improvement remains slow and expensive (Wei and Jackson 2016). Breeding programmes rely heavily on multi-stage field evaluation and phenotypic selection, reflecting both the crop’s biology and the historical limitations of genomic tools (Wei et al. 2022). Modern cultivars are highly polyploid and heterozygous, often exhibiting strong non-additive genetic effects (Yadav et al. 2021b). These features complicate inheritance, reduce the predictability of progeny performance, and make it difficult to translate observed clonal performance into reliable parental merit (Hoarau et al. 2022). Further constraints arise from the long breeding cycle, the large spatial footprint of trials, clonal propagation, and the need to evaluate genotypes over multiple crop cycles (plant and ratoon crops) and environments (Wei et al. 2022).

Genomic selection (GS) has emerged as a powerful approach in many crops and livestock to increase selection accuracy and shorten the time to decision by predicting genetic merit from genome-wide markers (Meuwissen et al. 2001, 2016). In sugarcane, GS has progressed from proof-of-concept to routine use in some advanced clonal evaluation pipelines. However, its operational use remains concentrated in later stages, particularly among elite clones in multi-environment trials (SRA 2025b). This restriction limits the overall impact of genomics because the greatest opportunities for accelerating genetic gain per unit time are typically earlier in the pipeline: family evaluation, parent breeding-value estimation, cross allocation, and early clonal selection (Crossa et al. 2017). Unlocking these gains requires addressing a set of interlinked biological and analytical challenges that are specific to sugarcane’s genome, trial structures, and crossing constraints.

Recent reviews on sugarcane genetics, breeding, and GS have largely focussed either on the performance of genomic prediction models and the molecular tools that enable them (Yadav et al. 2020; Hoarau et al. 2022; Jackson et al. 2022), or on broad overviews of genomics, biotechnology, and emerging breeding technologies (Meena et al. 2022; Lu et al. 2024; Srithawong et al. 2026), with programme-level accounts describing operational pipelines in detail (Wei et al. 2022). What remains less explicitly synthesised is how the statistical structure of multi-stage trial data shapes the genomic decisions that ultimately drive genetic gain.

In contrast to reviews focussed on individual components, we present a decision-centred synthesis of the sugarcane breeding pipeline that integrates multi-stage trial systems, genomic prediction, cross prediction, and mate allocation within a unified framework. This perspective reframes existing statistical and genomic methods in terms of their roles in breeding decisions, explicitly linking biological constraints to the analytical and operational choices available to breeders (Crossa et al. 2017; Cullis et al. 2020; Wei et al. 2022). Building on this foundation, we highlight opportunities for more systematic, data-driven breeding strategies.

This review is organised around three aims: (i) improving estimation of family and parental performance from early-stage trials, (ii) integrating pedigree and genomic information in mixed-model frameworks that reflect the biology and design of sugarcane trials, and (iii) moving beyond selection of individuals towards prediction and optimisation of crosses, including mate allocation under operational constraints. Sects. "Conventional sugarcane breeding context: history, traits, pipeline, and decision points" and "Biological constraints: genome complexity, trait architecture, and non-additive effects" establish the breeding context, decision points, and biological constraints imposed by sugarcane’s genome and genetic architecture. Sect. "Trial structure and statistical challenges across breeding stages" addresses the first aim by examining the statistical structure of family and clonal trial data, including family plot means, spatial and competition effects, and genotype-by-environment interaction; Sect. "Genetic gain and the leverage points in sugarcane improvement" links these analytical choices to genetic gain. Sects. "Genomic resources and genotyping in sugarcane" and "Genomic selection in sugarcane: models, performance, and limitations" address the second aim, covering genomic resources, genotype representation, allele dosage, and genomic prediction models. Sect. "Beyond selection: genomic cross prediction and mate allocation" addresses the third aim by developing cross prediction and mate allocation as decision-support tools under biological and operational constraints. Sect. "Future progress in sugarcane quantitative breeding" integrates these threads to identify priorities for more systematic, data-driven breeding. While the examples and terminology draw strongly from the Australian programme (Sugar Research Australia; SRA) and comparable systems, the methodological themes generalise to many sugarcane breeding pipelines globally.

## Conventional sugarcane breeding context: history, traits, pipeline, and decision points

### Germplasm and hybridisation in the Saccharum complex

Modern sugarcane cultivars reflect extensive hybridisation and introgression within the *Saccharum* complex. Wild species such as *S. spontaneum* and *S. robustum* and cultivated species such as *S. officinarum* have contributed to modern germplasm, alongside related genera including *Erianthus* and others (Heinz 1987). The historical transition from “noble canes” (*S. officinarum*) to interspecific hybrids improved disease resistance and adaptation while increasing genomic complexity. Nobilisation, involving interspecific crossing and backcrossing to *S. officinarum* to recover sucrose-related alleles, delivered landmark cultivars and contributed to the relatively narrow global genetic base of modern cultivars, many of which descend from a small number of key ancestors (Bremer 1961). In Australia, germplasm encompasses domestic elite parents, international introductions, basic germplasm collections, and targeted introgression programmes designed to broaden diversity and introduce novel traits (Piperidis et al. 2008; Wei et al. 2022).

This history has direct analytical consequences. A narrow genetic base can increase relatedness and linkage disequilibrium (LD) (Jannoo et al. 1999), which may support genomic prediction within closely related breeding populations over short prediction horizons but can constrain long-term gain and increase inbreeding risk if mate allocation is not managed carefully (Yadav et al. 2023). Introgression can introduce beneficial alleles but also disrupt favourable allele combinations and exacerbate segregation and non-additive effects, raising the value of cross prediction and stability analysis (Wang et al. 2013; Gouy et al. 2015; Piperidis et al. 2020).

### Economic importance and production context

Sugarcane is cultivated widely and contributes substantially to global sugar supply and bioethanol production, with Brazil, India, and China among the major producers and a smaller but commercially important industry in Australia (FAO 2025). Beyond sucrose, cane biomass supports renewable energy generation, paper and packaging, and other value streams, including emerging applications in advanced materials and environmental technologies (Santos et al. 2020; Mod et al. 2024). In Australia, production is concentrated in Queensland, with a smaller industry in northern New South Wales, and spans high-rainfall tropical and subtropical regions as well as irrigated production areas. Industry profitability reflects both agronomic performance and market volatility. Pressure from climate variability, changing seasonal rainfall and temperature patterns, and rising input costs increase the value of improved varieties and more efficient breeding systems (ABS 2025; Canegrowers 2025).

From a breeding perspective, this context reinforces three priorities. First, trait improvement must be multi-dimensional and include yield, sugar content, fibre, ratooning ability, disease resistance, and adaptation. Second, environment is not a single factor but a composite of region, season, crop cycle, and management. Third, breeding decisions must be made under resource constraints, so statistical efficiency and decision timing are important alongside genetic potential.

### Commercial traits in the Australian production system

Commercial cane systems typically evaluate (i) tonnes of cane per hectare (TCH), capturing biomass yield, (ii) commercial cane sugar (CCS), which estimates recoverable sucrose, and (iii) fibre percentage, reflecting milling constraints and bioenergy potential. TCH is influenced by stalk number, height, diameter, and stand density; CCS is derived from juice quality measures (BSES 1984). Ratooning ability is the capacity of a plant to re-shoot productive ratoon crops after harvest, and it contributes substantially to whole-cycle value because repeated annual yields from the same stool reduce establishment costs (Xu et al. 2021). Sugar yield is often expressed as tonnes of sugar per hectare (TSH), typically calculated from TCH and CCS, highlighting the need for simultaneous improvement. Reported genetic correlations between yield and sugar content are often weak or unfavourable, complicating selection decisions and cross design (Shahi et al. 2025). Fibre adds an additional axis because it can be penalising for milling but desirable for bioenergy objectives (Islam et al. 2021b). The multi-trait nature of commercial value therefore makes single-trait optimisation inadequate and heightens the importance of modelling trade-offs and stability in selection and cross design.

### The breeding pipeline and decision points: recurrent selection adapted to clonal crops

**Fig. 1:**
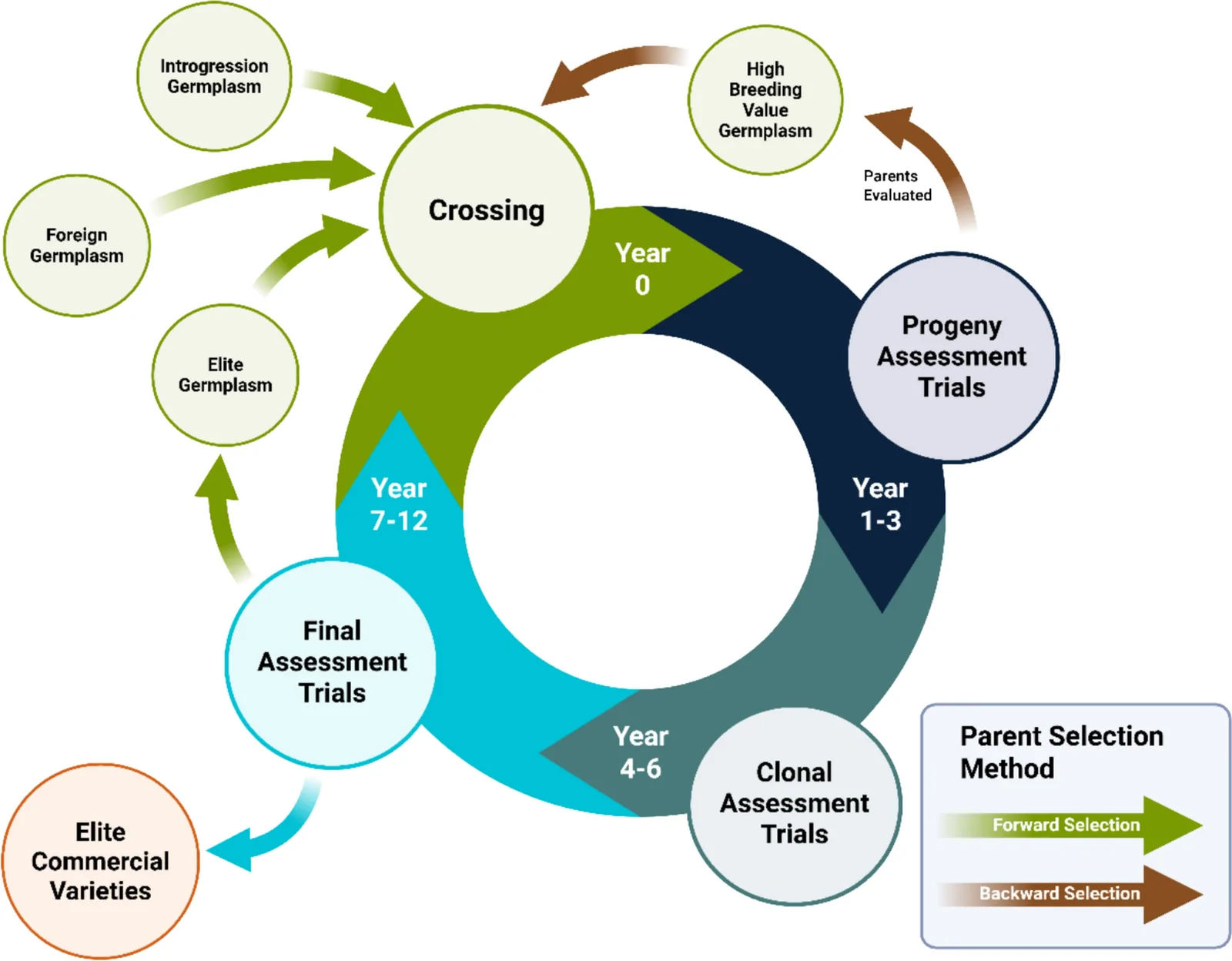
Recurrent selection pipeline used in the *Sugar Research Australia (SRA)* breeding programme. Controlled crossing generates true seed that enters PATs for family ranking and backward selection of parents using family plot means. Selected individuals advance to CATs for early clonal selection under limited replication and subsequently to FATs for multi-environment evaluation*.* Superior clones may be released commercially, or recycled into the parental pool through forward selection*.* Created in BioRender. Rigby, A. J. (2026) https://BioRender.com/4gx8j0t*.* PATs = Progeny Assessment Trials; CATs = Clonal Assessment Trials; FATs = Final Assessment Trials

Sugarcane breeding operates as a modified recurrent selection system adapted to clonal propagation and long cycle times. In the Australian programme, annual crossing produces true seed; progenies are evaluated through successive stages, and a small fraction of elite clones are released commercially or recycled as parents after extensive testing. Early stages focus on families in progeny assessment trials (PATs), followed by early-generation clonal assessment trials (CATs), and advanced multi-environment final assessment trials (FATs) (Fig. 1). The operational pipeline spans roughly a decade or more from cross to release, with overlapping generations and opportunistic recycling of parents rather than sharply delineated cycles (SRA 2024; SRA 2025a).

Parent selection in sugarcane blends backward selection, where progeny performance is used to infer parental breeding values, and forward selection, where high-performance clones that advance through clonal evaluation are considered as candidate parents (Fig. 1). Backward selection depends on analysing PAT data across years and regions, often using pedigree relationships to estimate parental breeding values (Atkin et al. 2009). Forward selection is constrained because clonal performance captures total-genetic value, including non-additive effects that may not transmit reliably to progeny (Wei et al. 2022). Practical crossing is further constrained by flowering synchrony, pollen viability, compatibility, and seasonal conditions (Moore and Berding 2014). Decision-support systems such as the Sugarcane Plant Improvement Database (SPIDNet) rank feasible crosses based on breeding values, relatedness, disease ratings, compatibility, and seed inventories (Wei et al. 2022). The outcome is an inherently constrained mate-allocation problem rather than an unconstrained optimisation task. These constraints motivate models that can predict cross performance under sparse and opportunistic mating designs and can incorporate compatibility and flowering phenotypes (SRA 2024).

**Fig. 2:**
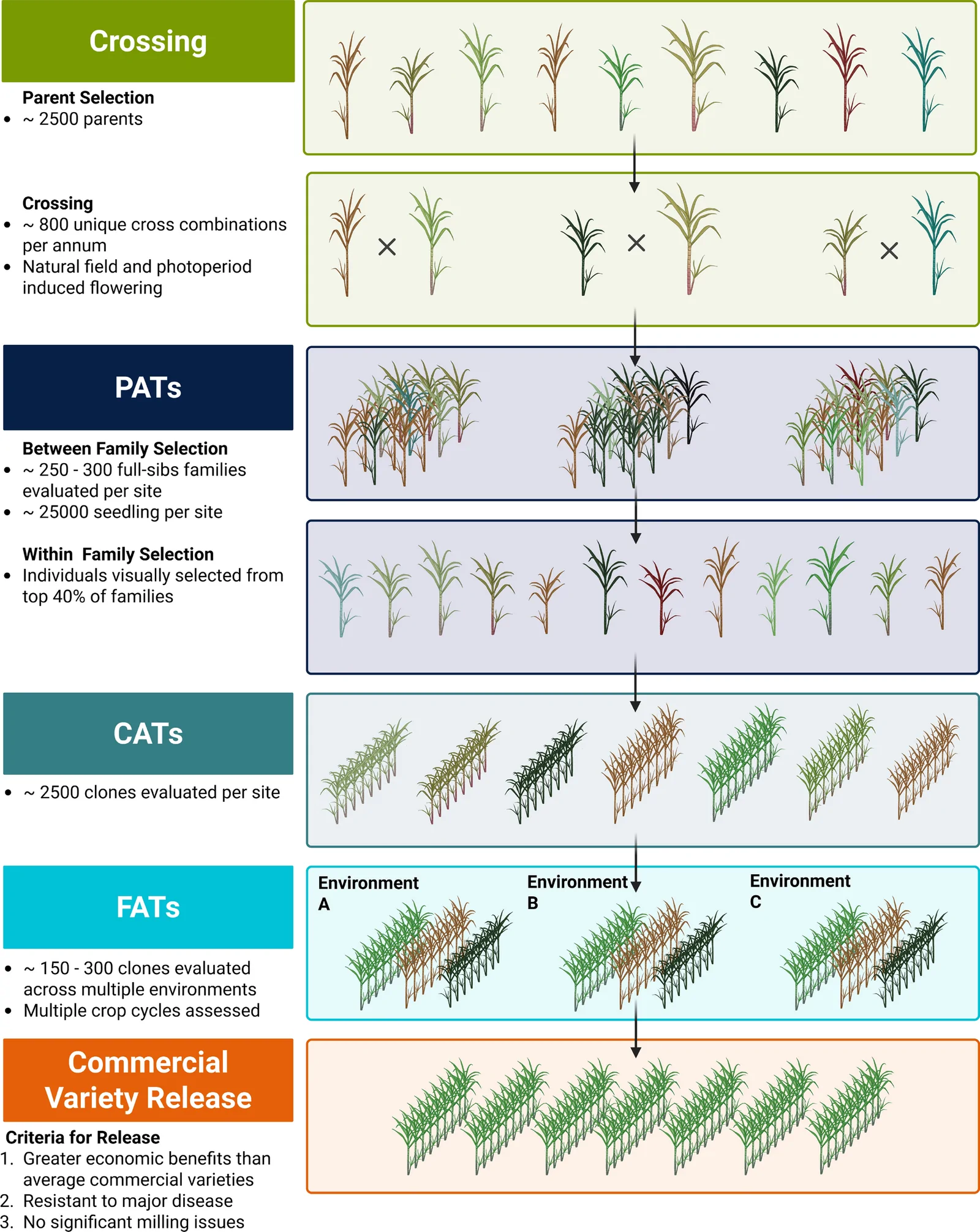
Selection decision points in the Australian sugarcane breeding programme. Breeding begins with parent selection and controlled crossing, followed by PATs where between-family selection is based on plant cane performance and within-family selection is conducted visually in the first-ratoon crop (1R) without formal measurement of ratooning ability. Individuals from promising families advance to CATs, and then progress to multi-environment FATs, where plant and multiple ratoon crops are evaluated from which superior genotypes are identified for commercial variety release and possible recycling as parents. Created in BioRender. Rigby, A. J. (2026) https://BioRender.com/acm5s7x. PATs = Progeny Assessment Trials; CATs = Clonal Assessment Trials; FATs = Final Assessment Trials

This structure creates a central statistical challenge: early-stage decisions determine which genetic variation persists to later stages, yet early-stage phenotyping is less precise and often based on aggregated family means. Later-stage phenotyping is more precise, but it occurs after strong selection has already reduced diversity (Wei et al. 2022). These stages can be expressed as a small set of recurring decisions, including which crosses to make, which families to advance, which clones to retain under noisy early testing, and which elite clones to release and recycle (Fig. 2). Importantly, breeding programmes worldwide converge on the same underlying decision points, regardless of operational differences (Stringer et al. 2011a; Zeni Neto et al. 2013; Mbuma et al. 2017).

At present, PAT, CAT, and FAT analyses are conducted as separate statistical problems. However, there is no routine statistical information sharing across selection stages. Information passes between selection stages almost entirely through selection decisions. The same parents appear as contributors to PAT family means, as clonal entries, and as check clones in clonal trials; yet their parental breeding values are not refined by a model that considers all these contributions. This review is structured around these decision points and evaluates how statistical and genomic tools most relevant to accurate selection can contribute to moving reliable selection earlier in the pipeline.

## Biological constraints: genome complexity, trait architecture, and non-additive effects

### Polyploidy, aneuploidy, and inheritance

Sugarcane is a highly polyploid and frequently aneuploid crop (Henry and Kole 2010). In this context, aneuploidy means that chromosome copy numbers may vary among homoeologous groups and genomic regions, so local ploidy is not necessarily uniform within or among cultivars (Garcia et al. 2013). Modern cultivars often have on the order of 100 to 130 chromosomes and are derived from interspecific hybridisation among ancestral lineages. Species within the Saccharum complex vary widely in chromosome numbers and ploidy, and chromosome transmission in hybrids can deviate from classical Mendelian expectations, including through unreduced gametes generated by restitution mechanisms (Piperidis et al. 2010; Hermann et al. 2012). These features complicate genetic interpretation, limit the applicability of diploid assumptions, and make genotype calling and variance partitioning challenging (Bourke et al. 2018).

Modern sugarcane combines features of autopolyploid and allopolyploid inheritance, with evidence for predominantly random pairing, alongside preferential chromosome pairing among subsets of chromosomes (Grivet et al. 1996; Hoarau et al. 2001; Jannoo et al. 2004; Aitken et al. 2007). Diploid-like segregation can occur at single-dose loci, enabling simplified analyses based on marker presence or absence rather than full allele dosage (Aitken 2022), a strategy widely used in GS studies (Deomano et al. 2020; Hayes et al. 2021; Yadav et al. 2021b). While operationally useful, diploidisation discards allele-dosage information and may fail to represent dosage-dependent additive and non-additive effects, as discussed further in Sect. "Diploidisation versus dosage-aware genotypes" (Batista et al. 2022; Yadav et al. 2024).

### Genetic architecture of key traits

Cane yield, sugar yield, and their component traits are polygenic and strongly influenced by genotype-by-environment (G×E) interactions (Pinto et al. 2010; Singh et al. 2013). Quantitative trait loci (QTL) studies often identify small-effect loci explaining a modest fraction of phenotypic variance, with evidence for antagonistic pleiotropy and trade-offs between yield and sugar traits (Jackson 2005; Aitken et al. 2008; Pinto et al. 2010). Sucrose-related traits are also polygenic and marker effects may differ across crop cycles and maturity stages (Saavedra-Díaz et al. 2024). Epistatic marker interactions have also been reported, although their limited consistency across crop cycles makes their predictive value uncertain (Alwala et al. 2009). Fibre content is also genetically complex, with relationships to sucrose traits that can be unfavourable and relationships to yield that can vary by population and environment (Islam et al. 2018, 2021b). This architecture supports genome-wide prediction models that can handle many small effects and G×E, while non-additive effects should be included only where the available data can estimate them reliably.

### Combining ability, heterosis, and non-additive variance

Several studies report substantial non-additive genetic variance in sugarcane, particularly for cane yield and related biomass traits (Mendes de Paula et al. 2020; Zhou 2020; Yadav et al. 2021b). In classical quantitative genetics, additive effects underpin long-term response to selection, while dominance and epistasis contribute to cross-specific performance and heterosis. Combining ability frameworks partition these contributions into general combining ability (GCA), which describes a parent’s average performance across its crosses, from specific combining ability (SCA), which describes the deviation of a particular cross from that expected from parental GCA (Falconer and Mackay 1996). GCA is associated mainly with additive effects and SCA with non-additive effects, although this correspondence is not exact. In sugarcane, the correspondence between GCA and additive breeding value is complicated by polyploid inheritance, allele dosage, and the set of mating partners used to estimate GCA. Importantly, crosses between parents with high breeding values may not necessarily yield the highest-performing families when cross-specific effects are important (Wei et al. 2013).

In operational breeding, “proven crosses” are repeatedly used because they have produced elite progeny, implicitly capturing a mixture of parental additive merit, compatibility, and potentially favourable non-additive interactions (Kimbeng and Cox 2003; Stringer et al. 2011a). The downside is that heavy reliance on proven crosses can reduce exploration of new combinations and constrain long-term gain and diversity. The relevance of non-additive effects also differs across stages of the breeding pipeline. For clonal evaluation, the total-genetic value, including dominance and epistatic deviations, is critical, as the genotype is fixed and propagated intact. For parent selection, additive breeding value remains central because it represents the component expected to be transmitted across progeny (Falconer and Mackay 1996). For cross prediction, however, the expected family means may also include cross-specific non-additive effects represented by SCA (Zhou 2020; Yadav et al. 2023). Increasing family size improves the precision of the estimated cross mean but does not force its expected SCA or dominance component to zero.

Taken together, these considerations indicate that non-additive variance is a substantial component of genetic architecture in sugarcane and cannot be ignored, but its practical importance is stage dependent. Current evidence supports the inclusion of non-additive effects to improve clonal selection accuracy for some traits, whereas their contribution to cross prediction remains uncertain, limited by data structure and allele-dosage resolution, and represents an important area for future research. For this reason, we treat non-additive effects in a stage-specific manner in the sections that follow, distinguishing their role in genetic gain (Sect. "Genetic gain and the leverage points in sugarcane improvement"), genomic prediction models and dosage-aware genomic relationship matrices (GRMs) (Sect. "Genomic resources and genotyping in sugarcane" and "Genomic selection in sugarcane: models, performance, and limitations"), and cross prediction and mate allocation as decision tools (Sect. "Beyond selection: genomic cross prediction and mate allocation"), rather than attempting a single unified treatment.

## Trial structure and statistical challenges across breeding stages

### Family trials: plot means, progeny sampling, and scaled Mendelian sampling

Family trials such as PATs are the first selection stage in many programmes and serve a dual purpose. They support selection of superior families and estimation of parental breeding values from progeny performance (SRA 2025a). In SRA PATs, each plot contains multiple unique seedlings from a full-sib family, and families may be represented by multiple plots, each with a different set of individuals (Fig. 2).

This design introduces Mendelian sampling variation both among seedlings within each plot and among plots representing the same cross, because family plots are repeated samples from the same cross population rather than true biological replicates (Stringer et al. 2011a; Atkin 2012). If family plots are analysed as repeated observations of a single genetic entity without accounting for within-family sampling variation, variance partitioning can be distorted and error variance may be inflated, reducing estimated heritability and compromising selection accuracy (Searle et al. 1992; Piepho et al. 2008).

In a plot mean of many offspring, this within-family sampling variance is reduced by averaging, but it is not eliminated unless the number of offspring is effectively infinite and sampling is unbiased (Falconer and Mackay 1996). Consequently, family-mean phenotypes require an appropriate modelling approach that reflects the scaled Mendelian sampling variance associated with the mean of sampled full sibs when genetic relationships are modelled. The adjustment is particularly important in animal-model parameterisations where plot means are treated as pseudo-individual observations to infer underlying genetic effects, especially when plots contain relatively few progenies (Cullis et al. 2014).

### Spatial heterogeneity and competition effects

Single-row plot designs used in family and early clonal trials are sensitive to spatial heterogeneity and interplot interference (Stringer et al. 2011b; Atkin 2012). A major source of bias arises from competition between neighbouring plots. Where the effect of a neighbouring genotype has a heritable component, it can be modelled as an indirect genetic effect (IGE) so the observed phenotype reflects both the genotype’s own performance (direct effect) and the influence of neighbouring genotypes (Sakai 1955; Griffing 1967; Bailey and Desjonquères 2022). Neighbour effects are genetically structured and therefore cannot be treated as independent residual variation. If ignored, competition effects can distort estimates of direct genetic merit and alter genotype rankings (Muir 2005). This is especially relevant for yield traits such as TCH, because performance in heterogeneous single-row trials may differ from performance in commercial single-variety stands (Stringer et al. 2011b). In the absence of explicit competition modelling, there is a risk that selection favours genotypes that are highly competitive in single-row trials but less productive under single-variety commercial stands. However, competition effects can be confounded with spatial trends, plot size, and local environmental variation, so direct and indirect genetic effects are estimable only when the trial layout provides sufficient neighbour connectivity and the model is not over-parameterised. Modelling competition has been explored in sugarcane at the single-trial level but extending these concepts into multi-environment frameworks and its effect on selection decisions remain important research gaps.

### Confounding of environment, year, and crop cycle

Sugarcane trial data often involve multiple crop cycles (plant cane, first ratoon, second ratoon) evaluated across years and locations. Crop cycle and year can be confounded when a genotype is observed in different cycles under different seasonal conditions (Jackson et al. 2022). As a result, “environment” cannot be treated simply as location or region alone. For both families and clones, interactions may occur with location, year, crop cycle, and management, and these components can contribute differently depending on trait and stage. Studies have reported significant family by location and family by location by crop-year interactions for yield components, and with less consistent evidence for family by crop-year effects (Jackson et al. 1995; Mbuma et al. 2020). In clonal trials, environmental covariates such as harvest age, temperature, and water availability may help explain G×E beyond geographic location alone, although their importance varies among traits and datasets (Ramburan et al. 2011; Natarajan et al. 2020; Todd et al. 2025). These findings motivate higher-resolution environment definitions and modelling strategies that can separate seasonal and management drivers from geographic structure. However, year and crop cycle cannot be fully separated when they are structurally confounded, so interpretation should remain consistent with the trial design.

### Mixed models for multi-environment trials: FA models, stability, and interpretability

Multi-environment trial (MET) analysis in plant breeding has progressed from fixed-effect interaction models such as additive main effects and multiplicative interaction (AMMI) (Gauch 1992) and genotype plus genotype-by-environment interaction (GGE) biplot analysis (Yan et al. 2000) to mixed-model approaches that can handle unbalanced data and incorporate genetic relationships. Factor analytic (FA) models are now widely used as parsimonious covariance structures to model G×E interaction, particularly when the number of environments is large and a fully unstructured covariance matrix would require too many parameters (Smith et al. 2001). FA models provide a practical balance between flexibility and parsimony (Kelly et al. 2007), and in sugarcane, they have been adopted in analyses of family and parent performance (Atkin et al. 2009; Atkin 2012). However, FA models can be difficult to interpret biologically because latent factors do not directly identify environmental drivers, and single order FA models can capture scale interactions while missing cross-over interactions that drive re-ranking and instability, potentially leading to overly optimistic inferences about broad adaptation (Smith and Cullis 2018; Smith et al. 2021b, 2025).

Beyond modelling G×E interactions, a range of FA-based selection tools have been developed to support interpretation and selection decisions from fitted FA models (Smith and Cullis 2018). More recently, interaction class (iClass) approaches have been proposed to classify environments based on their genetic correlation structure, enabling the delineation of environment groups with comparable genotype responses (Smith et al. 2021b, 2025). While iClasses offer a valuable, data-driven framework for summarising variety performance, their definition is conditioned on the specific MET, trait, and FA model. In sugarcane’s multi-stage pipeline, frequently unbalanced early-stage trials, and multi-trait selection objectives imply that care will be needed in interpreting environment classes.

An additional operational limitation arises when environments are defined too broadly (for example, by aggregating multiple years within a region). Such choices can mask important year-to-year variability, hinder assessment of stability across seasons, and limit the capacity to identify families or clones that perform consistently under variable climate conditions (Oliveira et al. 2020; Smith et al. 2021b). Current computing capacity makes it feasible to compare alternative environment definitions and higher-order FA models, but additional complexity should be retained only when it improves model fit, prediction, or breeding decisions. Choosing an appropriate MET model directly affects how well we can identify broadly adapted or specifically adapted clones and therefore impacts selection decisions for stability versus performance.

### Single-stage and two-stage analyses: weighting, covariance, and double shrinkage

In MET analyses, two general modelling strategies are used: single-stage analyses and two-stage analyses. A single-stage approach jointly models trial-level spatial effects and across-environment genetic effects within a single mixed model. In contrast, a two-stage approach first derives adjusted means for each environment and then uses these means in a subsequent across-environment analysis. Despite the advantages of single-stage analyses, two-stage approaches remain common in breeding analytics because they separate trial-level spatial modelling from across-environment genetic analysis, thereby simplifying the analysis pipeline and reducing computational demands.

In the first stage of a two-stage analysis, genotypes are typically fitted as fixed effects within each trial to obtain adjusted means (best linear unbiased estimates; BLUEs) and their associated error variance–covariance matrix. This matrix quantifies the precision of the adjusted means and accounts for correlations among estimation errors arising from the experimental design, missing data, and spatial adjustment. Importantly, it is distinct from the pedigree- or marker-based genetic covariance among genotypes. These BLUEs are then used as response variables in the second analysis, in which genetic and G×E effects are typically modelled as random effects. Fitting genotypes as fixed in stage one provides unshrunk, trial-adjusted means for this subsequent analysis. If only the adjusted means are carried forward, information about their differing precision and correlated estimation errors is lost. Using inverse-variance weights based only on the diagonal elements of this matrix accounts for unequal precision but ignores correlations among estimation errors (Gogel et al. 2018; Verbyla 2023).

Genotypes may also be fitted as random effects in the first stage, yielding best linear unbiased predictions (BLUPs). Unlike BLUEs, BLUPs are shrunk towards the population mean, and their direct use as stage-two response variables can lead to double shrinkage and biased inference (Holland and Piepho 2024). Consequently, first-stage BLUPs should not generally be used as ordinary responses in stage two unless their shrinkage and associated uncertainty are explicitly accommodated, for example through de-regression or an equivalent approach (Garrick et al. 2009; Holland and Piepho 2024).

These distinctions are particularly important in sugarcane breeding, where early-stage trials are often unbalanced, spatially adjusted, and only weakly connected across environments. While single-stage analyses provide a statistically attractive framework by modelling all sources of variation simultaneously, correctly weighted two-stage analysis that carries forward unbiased genetic effects and their associated uncertainty remains a valid and practical alternative for large operational datasets (Piepho et al. 2012; Gogel et al. 2018; Verbyla 2023).

## Genetic gain and the leverage points in sugarcane improvement

Genetic gain can be expressed through the breeder’s equation (Lush 1943), as a function of selection intensity, selection accuracy, additive genetic variation, and breeding-cycle length. In sugarcane, all four components can constrain genetic progress, but breeding-cycle length is the most significant limitation. Several years of field evaluation commonly separate crossing from the identification and recycling of superior parents, while commercial cultivar release requires substantially longer (Hoarau et al. 2022; Wei et al. 2022). Genomic approaches have the potential to increase selection accuracy and accelerate genetic gain by enabling selection decisions earlier in the breeding cycle (Goddard 2009; Desta and Ortiz 2014; Meuwissen et al. 2016; Yadav et al. 2020; Mahadevaiah et al. 2021; Werner et al. 2023). However, gains from improved prediction accuracy depend on whether decisions are made early enough to influence which families and clones proceed and which parents are recycled. This is why restricting GS to FATs may deliver local benefits in selection efficiency but is likely to deliver smaller system-wide gains compared with strategies that support earlier parent and crossing decisions (Hayes et al. 2021; Voss-Fels et al. 2021).

Estimates of heritability and selection accuracy are also sensitive to modelling choices. Mischaracterising repeated family plots as biological replicates, failing to account for competition or spatial heterogeneity, and defining environments too coarsely can bias variance-component estimates and reduce the accuracy of genetic evaluation (Falconer and Mackay 1996; Hoarau et al. 2022). Similarly, inadequate partitioning of additive and non-additive variation can confound breeding-value estimation and lead to suboptimal parent and cross decisions (Yadav et al. 2021b, 2023). Methodological improvements in mixed-model analysis and trial interpretation therefore influence realised genetic gain through their effects on selection accuracy and decision-making throughout the breeding pipeline.

Early decisions in PAT and early clonal stages determine which crosses, families, and segregants remain available for intensive testing and parent recycling, as outlined in Sect. "The breeding pipeline and decision points: recurrent selection adapted to clonal crops". Improving analytical efficiency at the earliest stages can therefore increase selection accuracy while genetic diversity remains relatively broad and produce more reliable parental breeding values, family performance, and predicted cross means for genomic prediction and mate-allocation strategies (Cobb et al. 2019). However, the extent to which these analytical improvements increase realised annual genetic gain at whole-system level has not yet been formally quantified in sugarcane breeding and remains an important research gap.

## Genomic resources and genotyping in sugarcane

### Marker technology and operational platforms

Sugarcane genomics has progressed through phases of marker development from early markers (isozymes, restriction fragment length polymorphisms; RFLPs) (Wood 1987; Burnquist 1991; Grivet et al. 1996) to polymerase chain reaction (PCR)-based markers (random amplified polymorphic DNA; RAPD, amplified fragment length polymorphisms; AFLP, simple sequence repeats; SSR) (Silva et al. 1995; Hoarau et al. 2001; Aitken et al. 2005) and then to high-throughput array and sequencing-based single nucleotide polymorphisms (SNP) platforms. Diversity arrays technology (DArT) enabled early high-throughput genotyping and supported QTL (Wei et al. 2010; Costet et al. 2012; Aitken et al. 2014) and association studies and was used in an early experiment assessment of GS in sugarcane (Gouy et al. 2013). SNP arrays, including the Affymetrix Axiom platforms, have become central to many applied studies and some operational pipelines (Aitken et al. 2016). These SNP arrays are robust and scalable but are relatively expensive and become less informative over time if the breeding population evolves and marker polymorphisms change, particularly when relying heavily on single-dose markers. Fixed panels are inevitably shaped by the germplasm used for SNP discovery and tend to capture the allele frequency spectrum of their discovery sets (Aitken et al. 2016; Rasheed et al. 2017).

Critically for GS, the decline in informativeness has two mechanistic pathways. First, as the breeding population diverges through selection and drift, the allele frequencies at discovery-set SNPs shift, and some previously polymorphic sites approach fixation, reducing the number of informative, segregating SNPs available to construct GRMs. Second, the LD phase between markers and QTL can drift across generations, so a marker that reliably tagged a QTL allele in the training population may no longer do so in the prediction population (de Roos et al. 2008; Sallam et al. 2015). The practical consequence is that genomic prediction accuracy decays across cycles unless models are retrained with contemporary phenotypes and genotypes. As long as marker density and LD coverage remain sufficient, the loss of individual polymorphisms is largely buffered by neighbouring SNPs in strong LD; the concern becomes acute when a substantial fraction of markers approach fixation or when the target population has diverged substantially from the discovery panel. Genotyping-by-sequencing (GBS) offers flexibility and potentially lower per-sample cost (Pérez-Enciso et al. 2015), but poses challenges in sugarcane due to genome size, ploidy, dosage calling, uneven read depth, and missing data (Scheben et al. 2017). While GBS can support SNP discovery and may be suitable for some research applications (Yang et al. 2017; Gutierrez et al. 2018), operational prediction pipelines require consistent, high-quality genotype calls and careful quality control to avoid reducing prediction accuracy (Manimekalai et al. 2020).

### Diploidisation versus dosage-aware genotypes

Most GS studies in sugarcane have simplified marker data to pseudo-diploid classes that collapse multiple heterozygous dosage states into a single class (Hayes et al. 2021; Voss-Fels et al. 2021; Yadav et al. 2021b; Gonçalves et al. 2025), motivated by the prevalence of single-dose markers that segregate in a diploid-like manner (George and Aitken 2010; Aitken 2022). This strategy has been operationally successful, but it inevitably collapses information on allele copy number and cannot fully represent the complexities of inheritance expected in a highly polyploid crop. This section reviews the evidence on when and how dosage-aware representations improve prediction, introduces the distinct complication of aneuploidy, and draws practical conclusions for current and future practice.

#### Dosage-aware SNP calling

Discrete dosage calling in polyploids requires assigning each individual to one of (ploidy + 1) dosage classes (nulliplex, simplex, duplex, … up to hexaplex or beyond) at each SNP. Software tools such as *polyRAD* (Clark et al. 2019), *updog* (Gerard et al. 2018), *SuperMASSA* (Serang et al. 2012), and *fitPoly* (extension of the earlier *fitTetra* approach) (Voorrips et al. 2011) use model-based approaches that incorporate sequencing depth, expected dosage ratios, and population allele frequency priors. Caller choice matters because alternative algorithms may produce different dosage estimates and consequently different downstream results. Njuguna et al. (2023) showed that different calling pipelines (*polyRAD* versus *updog*) yielded meaningfully different genome-wide association studies (GWAS) signals in the same autopolyploid Miscanthus and blueberry datasets, and that per-locus prediction accuracy varied with both caller and read depth. This implies that the genotype-calling step is not a neutral preprocessing step but an analytical decision with downstream consequences for prediction accuracy and marker-trait associations. For sugarcane, where SNP arrays provide stable intensity data but often form near-continuous cluster bands rather than discrete clusters and are enriched for low-dosage markers, discrete dosage calling is especially challenging (Garcia et al. 2013; Yadav et al. 2024).

#### Continuous-genotype representation

One response to calling uncertainty is to abandon discrete dosage classes in favour of continuous-genotype representations, using posterior-mean dosage estimates or normalised array intensities as genotype values. Njuguna et al. (2023) showed in simulation that posterior-mean continuous dosages increased genomic prediction accuracy across a range of read depths compared to hard-called diploid or polyploid genotypes, primarily by reducing misclassification error. In sugarcane, Yadav et al. (2024) used normalised Axiom array intensities as continuous genotypes (*n* = 1,318 clones; ~ 26 K SNPs post-QC) and compared them to pseudo-diploid encodings. Continuous genotypes gave consistent but modest accuracy gains of approximately 5–7% for CCS and fibre percentage, while pseudo-diploid markers performed slightly better for cane yield (TCH). The authors attributed the limited benefit of dosage for TCH to the high proportion of simplex (single-dose) markers in the panel, which carry limited dosage information. Nonparametric kernels (reproducing kernel Hilbert space; RKHS, arc-cosine) performed best for TCH and CCS when combined with continuous genotypes, suggesting that continuous representations interact with model class in trait-specific ways. Researchers should therefore treat continuous genotypes as a potentially useful option, particularly for traits with evidence of dosage effects, rather than a universal improvement over pseudo-diploid encoding.

#### Polyploid-specific relationship matrices and dosage-aware prediction models

The theoretical framework for dosage-aware genomic prediction in polyploids was formalised for autotetraploids (Endelman et al. 2018) and extended to higher and mixed ploidy by Batista et al. (2022). Their framework derives additive and digenic dominance relationship matrices that correctly account for allele dosage across ploidy levels. In simulation scenarios featuring frequent multi-dose heterozygotes and strong dominance effects, dosage-aware models substantially outperformed diploidised encodings, in some scenarios yielding up to approximately 140% higher mean predictive abilities. However, in real sugarcane and sweet potato datasets where the frequency of heterozygous genotype classes is low (populations dominated by simplex or homozygous markers), dosage-aware models provided little or no advantage over diploid marker classification (Batista et al. 2022). This result is consistent with the analytical principle that dosage-aware models add value when informative dosage variation exists in the marker set; when the population is predominantly simplex and the distribution of heterozygous states is low, the additional complexity does not pay off. Lara et al. (2019) similarly found moderate improvements in autotetraploid *Panicum maximum* when dosage information was included, conditional on the proportion of multi-dose markers.

Taken together, these studies suggest a coherent practical pattern: dosage-aware models are likely to deliver meaningful gains when (i) multi-dose genotypes are common at loci affecting the trait, (ii) non-additive effects linked to dosage contribute to the target trait, and (iii) calling pipelines provide sufficiently accurate dosage information. The critical implication for cross prediction and mate allocation (addressed in Sect. "Beyond selection: genomic cross prediction and mate allocation") is that within-cross variance prediction, which depends on the distribution of allele dosage across candidate parents, will be systematically underestimated if dosage is incorrectly characterised. This is an area where the quality of dosage information has direct downstream consequences for breeding decisions beyond genomic prediction alone. As breeding cycles accumulate, parental backgrounds diversify, and dosage distributions at array loci drift further from discovery-panel conditions, the value of accurate dosage representation is likely to increase.

#### Aneuploidy: a distinct and underappreciated complication

Allele dosage is not the only complexity in sugarcane genotyping: aneuploidy adds an additional, distinct source of uncertainty. Modern sugarcane varieties display mixed ploidy and frequent aneuploidy, with per-locus ploidy estimates between six and fourteen copies per homoeologous group and higher local copy numbers at some loci (Garcia et al. 2013; McCord 2016; Healey et al. 2024). This means that the nominal ploidy of a variety does not define the local ploidy at any given SNP, and that accurately representing genetic variation requires joint estimation of local ploidy level and allele dosage at each marker (Garcia et al. 2013). The consequence for genomic analysis is that standard dosage models (which assume a fixed ploidy) may be misspecified at a substantial proportion of loci, introducing systematic errors in GRM estimation and in the modelled relationship between marker dosage and phenotype (Garcia et al. 2013; Batista et al. 2022). In clonal prediction, these errors contribute additional noise and potential bias in breeding-value and total-genetic value estimates over and above dosage calling uncertainty.

Aneuploidy further complicates cross prediction because it undermines our ability to specify segregation at marker loci. When parents differ in ploidy or copy number at a locus, the number of allelic copies transmitted and their meiotic behaviour no longer follow simple fixed-ploidy Mendelian expectations. As a result, the expected dosage distribution among progeny, and therefore predicted cross means and within-cross variance can only be approximated (Falconer and Mackay 1996; Garcia et al. 2013; Healey et al. 2024). This limitation is evident in current sugarcane mate-allocation frameworks, discussed further in Sect. "Beyond selection: genomic cross prediction and mate allocation", which simulate diploid inheritance to project parental genotypes into progeny performance. This diploid segregation is acknowledged as an approximation that becomes less accurate in regions of higher local ploidy (Yadav et al. 2023). Consequently, within-cross variance predictions for dosage-dependent traits may be biased, often downward, because the simplifying assumption of uniform ploidy collapses or ignores part of the potential variation in progeny dosage.

Aneuploidy therefore adds a distinct layer of uncertainty to both clonal and cross prediction. Addressing aneuploidy rigorously will require genotyping platforms and allele-calling algorithms that can jointly model local ploidy and allele dosage, a technically demanding but important long-term goal. In the meantime, cross-level predictions in mixed-ploidy sugarcane should be interpreted as useful for prioritising crosses, but with greater uncertainty in predicted cross means and within-cross variance than in systems with known and fixed ploidy.

### The R570 polyploid reference genome and its implications for breeding

Recent efforts have produced a highly contiguous polyploid reference genome of the modern interspecific hybrid R570 (Healey et al. 2024), providing a more complete representation than previous monoploid assemblies (Garsmeur et al. 2018; Souza et al. 2019; Shearman et al. 2022). The R570 assembly spans approximately 8.7 Gb and contains a comprehensive representation of the polyploid sequence space across approximately 12 chromosome copies, resolving individual haplotypes from both *S. spontaneum* and *S. officinarum* progenitor genomes.

This genome represents a valuable resource for advancing adaptive breeding in sugarcane, enabling more informative marker development, higher-resolution physical anchoring of QTL, and better resolution of single-copy loci such as the Bru1 brown-rust resistance gene (Healey et al. 2024). Beyond these immediate applications, the R570 assembly is particularly important for understanding and exploiting non-additive effects, especially epistatic effects which are found to contribute substantially to clonal performance for key traits such as TCH and CCS (Yadav et al. 2021b).

In this context, the R570 genome enables three distinct analytical advances that were previously difficult or impossible with monoploid or fragmented assemblies. First, by resolving haplotypes and chromosomes, the R570 genome provides a chromosome-scale scaffold on which SNP assays can be annotated within homoeologous groups. This provides a framework for epistasis models that can distinguish interactions within versus between haplotypes rather than between anonymous marker pairs (Jiang et al. 2018). However, short SNP-array probes that match multiple homoeologous or repetitive copies may remain ambiguous in existing array data (Aitken et al. 2016). Long-read sequencing and locus-specific marker development provide a pathway towards resolving these signals. Second, the resolved progenitor origins (*S. officinarum* versus *S. spontaneum* chromosome segments) allow epistasis to be tested in a genomic context that captures interspecific complementarity and heterosis (Bansal et al. 2012; Sang et al. 2022). In R570, detailed assignment of chromosome segments to each progenitor, together with recombinant chromosomes and enriched *S. spontaneum* resistance loci, enables prospective tests of whether epistatic interactions are concentrated within or between wild and domesticated haplotypes. Third, reliable physically anchored markers enable regional heritability analysis (Nagamine et al. 2012) and biologically informed SNP grouping (e.g. by gene pathway or chromosomal region), which can reduce the multiple-testing burden in epistasis mapping (Duroux et al. 2022) and support prediction models that incorporate prior genomic information (Tang et al. 2023). Aggregating SNPs into regions or functional units defined on the R570 scaffold can allow epistatic variance to be attributed to pathways or chromosome segments rather than isolated marker pairs, potentially making both detection and interpretation of interaction effects more robust in sugarcane (Healey et al. 2024).

It is important to note that while these epistatic analyses are now enabled, they remain largely prospective: to date, epistatic variance in sugarcane has been captured statistically (e.g. RKHS kernels accounting for higher-order interactions), but has not yet been attributed to specific interacting genomic regions (Yadav et al. 2021b). The R570 assembly provides the scaffold on which such work can now begin.

## Genomic selection in sugarcane: models, performance, and limitations

### Linkage disequilibrium, relatedness, and implications for prediction

Sugarcane breeding populations often show substantial relatedness and extended LD, reflecting both the founder bottleneck and the structure of recurrent selection and proven cross usage (Jannoo et al. 1999; Raboin et al. 2008; Yadav et al. 2021a). High and persistent LD can make genomic prediction more robust because markers can tag large haplotype blocks, and the unit of inheritance may be more effectively captured as haplotypes rather than individual SNPs (Raboin et al. 2008). This can also reduce the marker density required for stable prediction in some contexts (Shahi et al. 2025). However, strong relatedness can inflate cross-validation estimates if validation splits do not reflect forward prediction scenarios, and it can lead to diversity loss if selection decisions are made without explicit constraints on relatedness and inbreeding (Rodríguez-Ramilo et al. 2015).

### Foundational studies and current operational focus

Early work established that genome-wide markers could predict sugarcane clone performance for agronomic traits, supporting the feasibility of GS despite genomic complexity (Gouy et al. 2013). Subsequent studies evaluated model classes and marker densities relevant to Australian traits and populations (Deomano et al. 2020; Islam et al. 2021a; Yadav et al. 2021b; Chen et al. 2023; Yu et al. 2024) and compared genomic models to pedigree-based approaches (Hayes et al. 2021; Gonçalves et al. 2025). Integration of GS into advanced clonal trials has enabled more accurate selection among elite clones and may reduce time to decision in later stages (SRA 2025b). Yet the primary limitation remains the stage of application: if genotyping is confined to elite, highly selected clones, prediction models may not generalise well to early clonal stages and may provide limited leverage for parent and cross decisions (Clark et al. 2012; Bustos-Korts et al. 2016).

### Model classes: parametric, Bayesian, kernel, and machine learning

Across sugarcane studies, genomic best linear unbiased prediction (GBLUP) models (Deomano et al. 2020; Hayes et al. 2021; Yadav et al. 2021b, 2024; Chen et al. 2023; Inamori et al. 2024; Gonçalves et al. 2025; Shahi et al. 2025) and ridge regression best linear unbiased prediction (RR-BLUP) models (Gouy et al. 2013; Islam et al. 2021a; Shahi et al. 2025) remain common baselines because they perform well for highly polygenic traits under many small effects (Aitken et al. 2006; Singh et al. 2013). Bayesian methods (BayesA, BayesB, BayesR, Bayesian least absolute shrinkage and selection operator; LASSO) (Gouy et al. 2013; Deomano et al. 2020; Hayes et al. 2021; Shahi et al. 2025) allow heavier-tailed effect distributions and may help when a subset of loci contributes more variance. Kernel methods such as RKHS can capture some non-additive patterns without explicitly modelling dominance and epistasis matrices (Gouy et al. 2013; Deomano et al. 2020; Islam et al. 2021a; Yadav et al. 2021b, 2024; Shahi et al. 2025). Machine learning (ML) approaches, including random forests, support vector regression (SVR), and neural networks, have been explored with mixed outcomes; performance often depends on training-set size, trait heritability, and tuning, and gains over parametric baselines are not consistent (Islam et al. 2021a; Chen et al. 2023; Inamori et al. 2024; Shahi et al. 2025). Reported successes for specific disease traits may reflect non-additive architecture or particular marker-trait relationships, but there remains a risk of confounding non-additive signal with environmental noise if models do not align with the trial structure (Islam et al. 2021a).

These studies suggest that well-specified GBLUP/RR-BLUP and related extensions remain appropriate baselines for most commercial sugarcane traits, with more complex Bayesian, kernel, or ML models offering incremental, trait-specific improvements. To make these trade-offs explicit, Table 1 summarises the main model classes.

**Table 1:** Concise overview of major genomic prediction model classes.

Model class	Best condition/limitations in sugarcane context
GBLUP/RR-BLUP	Robust baseline approach for highly polygenic traits controlled by many small-effect loci. Computationally efficient and widely used but primarily captures additive genetic effects and may not capture non-additive effects explicitly
Extended GBLUP (additive + non-additive)	Useful when non-additive genetic effects contribute substantially to trait variation (e.g. TCH). Can improve prediction accuracy but requires larger datasets
Bayesian methods	Advantageous when traits are influenced by a mixture of small- and moderate-effect loci. For highly polygenic sugarcane traits, prediction accuracy is often similar to GBLUP while requiring greater computational effort
Kernel methods (e.g. RKHS)	Can accommodate nonlinear relationships without explicitly modelling covariance structures (GRMs). Predictive gains are generally trait- and population-dependent and are often modest
Tree-based machine learning	Can detect complex marker interactions with minimum model assumptions; however, prone to overfitting and often provide little improvement over parametric approaches
Support vector machines (SVM)	Nonlinear option for reduced marker sets; scaling and tuning are challenging with full SNP panels
Deep learning methods (e.g. convolutional neural networks; CNN)	Potential for complex interactions with very large datasets; currently rarely outperform well-specified models in sugarcane when training populations are limited

This table highlights the principal strengths and limitations of GS models in the context of trait improvement in sugarcane

### Integrating pedigree and genomics: single-step frameworks

Single-step GBLUP (ssGBLUP) integrates genomic and pedigree relationships in a unified H-matrix and can improve prediction when not all individuals are genotyped, a common constraint in operational programmes (Aguilar et al. 2010). In sugarcane, ssGBLUP has improved prediction for some traits and stages, and pedigree information can add value beyond genomics alone in some contexts (Hayes et al. 2021). Comparative work has also reported scenarios where pedigree-based prediction can outperform genomic-only prediction (Gonçalves et al. 2025), possibly due to selective genotyping and pedigree uncertainty in specific designs. These findings highlight that the benefits of genomics depend not only on the model but on the genotyping strategy, pedigree integrity, and representativeness of the training population.

### Accounting for non-additive effects

Given the importance of non-additive variance in sugarcane, extending prediction models to include dominance and epistatic GRMs or using kernels is a logical step (Yadav et al. 2021b). From a modelling perspective, this raises the question of how additive and non-additive components are represented and separated in genomic relationship structures when the goal is parent selection.

Results indicate that modelling non-additive effects can improve prediction accuracy for yield traits in some settings, but additive relationships often capture a large fraction of variance, with non-additive matrices redistributing variance components rather than adding entirely new information (Yadav et al. 2021b). This is consistent with the idea that in populations with strong relatedness and extended LD, additive relationship matrices can partly absorb non-additive variation (Muñoz et al. 2014), although this may come at the cost of interpretability and may not translate to robust breeding-value prediction. From a prediction perspective, this can be convenient, because additive models may achieve reasonable clonal prediction accuracy.

For parent selection, this modelling behaviour is problematic. When non-additive effects are implicitly absorbed within the additive term, the resulting breeding values no longer cleanly represent the additive effects that drive long-term response to selection. In such cases, models that appear to target breeding values are in fact estimating a mixture of additive and non-additive contributions, complicating interpretation and potentially biasing parent selection decisions. The potential benefit of explicit non-additive modelling therefore depends not only on the underlying biology but also on the quality of genotype calling. As discussed in Sect. "Diploidisation versus dosage-aware genotypes", any practical benefit from explicit non-additive GRMs depends on reliable allele-dosage information. Within this framework, additive-only models may remain adequate for clonal prediction, but explicitly modelling non-additive effects becomes important whenever clean estimation of additive breeding values is required for parent selection.

### Training-population design and prediction evaluation

Prediction accuracy depends strongly on training-population composition, relatedness between training and prediction sets, trait heritability, and effective population size (Goddard 2009; Clark et al. 2012; Seye et al. 2020; Melchinger et al. 2023). In sugarcane, repeated use of proven parents and crosses can increase relatedness and may stabilise prediction across years (Guo et al. 2013) but can also inflate cross-validation estimates if validation does not reflect the way GS is used in practice (Haile et al. 2021). The cost of genotyping is widely acknowledged as a practical limitation on expanding training sets into early-stage selection candidates.

More structured guidance on training-population design for sugarcane can be derived from first principles and cross-species studies. (i) Size vs accuracy: accuracy increases with training-population size but with strongly diminishing returns beyond a threshold that depends on trait heritability and effective population size (Goddard 2009); for highly polyploid species with complex GRM structure, this threshold may differ from diploid expectations. (ii) Parental representation: ensuring that key founders and the parents generating current target populations are represented in the training set is more critical than maximising total sample size; sparse but well-connected training populations can outperform larger, poorly connected ones (Hickey et al. 2014; Melchinger et al. 2023). (iii) Cross sampling: including progeny from many families, rather than deep sampling of few families, improves coverage of the allele frequency spectrum and avoids training-set relatedness inflation (Tolhurst et al. 2019). (iv) Validation design: estimated prediction accuracy depends on how closely the validation scenario matches the intended use of GS (Tolhurst et al. 2022).

In populations with strong relatedness and substantial LD, within-cycle random cross-validation often yields relatively high prediction accuracies because validation genotypes are closely related to the training set (Clark et al. 2012). From a classical GS perspective, this is not inherently problematic: GS was explicitly designed to predict the merit of untested but related genotypes in the same set of environments (Meuwissen et al. 2001). Moreover, selection environments in well-designed METs are not a random sample of all possible environments; they are deliberately chosen to represent the target population of environments (TPE) in which new varieties are expected to be deployed (Tolhurst et al. 2019). Under this view, we interpret high within-cycle accuracies as reflecting the fact that the training and validation sets share both genetic relationships and a carefully constructed environmental context, rather than automatically treating them as evidence of overfitting.

By contrast, forward prediction designs, such as training on earlier cohorts or years and predicting a subsequent cohort, pose a different and more demanding problem: they require prediction for both untested genotypes and unobserved environments. In this setting, estimated accuracies conflate the difficulty of predicting new genotypes with the difficulty of extrapolating G×E interaction into future environments (Tolhurst et al. 2022). For breeding decisions that focus on ranking genotypes within the range of environments represented in a MET, within-cycle cross-validation may provide a more direct assessment of how well GS supports selection among related candidates. In contrast, forward prediction across years may be more relevant when the objective is to anticipate performance in future, potentially novel environments, but it comes with additional uncertainty arising from unmodelled G×E interactions (Tolhurst et al. 2022).

## Beyond selection: genomic cross prediction and mate allocation

### Why cross prediction matters more in sugarcane than in many crops

Crossing capacity, field space, and evaluation time constrain how many parental combinations can be tested (Wei et al. 2022). Cross design decisions therefore shape the genetic variance entering the pipeline and influence both short-term gain and long-term diversity (Peixoto et al. 2025). Because non-additive effects are substantial and inbreeding depression is a recognised risk (Ferreira et al. 2005), cross performance cannot be reliably inferred from parental breeding values derived from PATs alone (Wei et al. 2013). This helps explain the continued emphasis on empirically successful (“proven”) crosses, though it also motivates prediction of untested crosses so that programmes can explore new combinations while managing risk.

### Concepts and methods: from tested crosses to untested crosses

Cross prediction extends genomic prediction by shifting the target from individual genetic merit to the expected mean and risk profile from a specific mating. Pedigree-based approaches exploit covariance between tested and untested crosses via relationship among parents (Bernardo 1996), and genomic relationships provide an analogue that captures realised relatedness (Maenhout et al. 2010). In sugarcane, this problem differs fundamentally from hybrid prediction in maize (Kadam et al. 2016) because progeny segregate widely and individual-level non-additive effects are not fixed (Kristensen et al. 2023; Werner et al. 2023). Consequently, cross prediction is most useful for ranking crosses for field testing and allocating crossing resources by estimating expected family mean, cross-level heterosis, and risks associated with relatedness and inbreeding rather than identifying individual progeny. In addition, predicting the expected within-family genetic variance of a cross is valuable, as families combining high mean performance with substantial segregation variance are more likely to produce exceptional clones (Allier et al. 2019; Wolfe et al. 2021).

Three related decision problems form a continuum from parent evaluation to constrained optimisation. Parent selection targets GCA and additive merit. This strategy increases the frequency of favourable alleles but can reduce heterozygosity and overlook heterosis when dominance and epistasis contribute to performance (Werner et al. 2023). Cross-level genomic prediction addresses these limitations by estimating the expected performance of specific parent combinations by capturing genetic complementarity and, where possible, non-additive effects (Bradshaw 2016). Mate allocation extends this further by formalising crossing decisions as an optimisation problem: it identifies the set of crossing pairs that maximises gain while adhering to constraints on relatedness and logistical feasibility (Kinghorn 2011; Akdemir et al. 2015; Gorjanc and Hickey 2018; Endelman 2024; Goda and Isik 2024). The framework can also be extended to account for non-additive effects and polyploid-specific considerations for managing inbreeding (Werner et al. 2023).

### The particular challenge of sugarcane family trials

Early-stage phenotypes are measured as plot means of unique seedlings, not as replicated genotypes (Wei et al. 2022). This structure reduces the observable Mendelian sampling variance and complicates estimation of cross-specific effects from family data alone (Falconer and Mackay 1996; Pedrozo et al. 2011; Mrode 2014). Accordingly, cross-prediction models must respect the family-mean observation unit and the sampling variance embedded in those means, as discussed in Sect. "Family trials: plot means, progeny sampling, and scaled Mendelian sampling". The contribution of non-additive genetic effects is also less straightforward to disentangle in this setting, because dominance and epistatic interactions expressed at the individual level are expected to be partly averaged when phenotypes are recorded as family means, making it more challenging to attribute variation in family performance to additive, dominance, and epistatic components.

The most direct path is a stage-integrated approach that links family means from PATs with individual clone data from CATs and FATs, integrating information across multiple stages of selection. However, design limitations in PATs, particularly the lack of replicated clones, must be addressed to fully realise their potential. Family means can inform parental breeding values and cross means across a broad set of matings, while clonal data inform within-family segregation, expression of non-additive effects, and stability across environments. Joint analyses must also address selection, because clones represent a biased sample of each family after strong culling (Smith et al. 2021a).

### Practical constraints and readiness for genomic mate allocation

Mate allocation in sugarcane is inherently constrained by flowering synchrony, pollen viability, and flowering propensity that can vary by season (Heinz 1987; Glassop et al. 2014; Moore and Berding 2014; Melloni et al. 2015). Decision support should therefore operate on the feasible set of crosses and, where possible, treat flowering and compatibility as heritable traits that can be predicted and managed through photoperiod treatments (Hayes et al. 2021). Seed set and seed inventory add an additional constraint because not all high-value crosses yield sufficient seed for reliable evaluation. Integrating seed store inventory with cross value ensures that breeders maintain adequate stores of high-priority seed while minimising opportunistic crosses of lower-priority combinations (Wei et al. 2022).

#### Technical readiness and implementation barriers

Full genomic mate allocation in sugarcane, incorporating within-cross variance prediction, non-additive effects, and inbreeding control, faces several linked technical barriers that distinguish it from simpler GS implementations. First, within-cross variance prediction requires accurate parental phasing and knowledge of the gametic covariance structure; at the approximately 12× chromosome dosage typical of modern sugarcane, phasing is not solved and remains a research-stage problem. Second, the usefulness criterion (UC = cross mean + selection intensity × predicted selection gain within the cross; Allier et al. 2019) requires both a reliable cross mean prediction and a reliable variance prediction; evidence from other crops indicates that variance estimates are substantially less accurate than mean estimates, particularly for small numbers of tested crosses (Lehermeier et al. 2017). Third, non-additive effects relevant to cross mean prediction (dominance/SCA) depend on dosage accuracy (Sect. "Genomic resources and genotyping in sugarcane"), which is currently limited in operational sugarcane panels. Fourth, mate allocation under inbreeding constraints requires an operational kinship/relatedness metric; for polyploids, traditional inbreeding coefficients may not align directly with parental kinship, motivating modified methods (Endelman 2024). Tools for mate allocation are available *(SimpleMating*, Peixoto et al. 2025; *AlphaMate*, Gorjanc and Hickey 2018) but have not been prospectively validated in an operational sugarcane pipeline.

#### Short-term operational options vs long-term priorities

Genomic estimated breeding-value (GEBV)-based parent ranking (GCA ordering) for cross mean prediction is feasible now and can reduce reliance on proven crosses by identifying high-GCA parents from within-pipeline data. Full within-cross variance prediction and polyploid-aware inbreeding control are longer-term research priorities that require improved phasing, dosage characterisation, and prospective validation before operational deployment. Together these considerations imply that crossing plans should be adaptive and updated as flowering and seed outcomes become known.

### Lessons from other crops and relevance to sugarcane

Genomic cross prediction has matured in maize (Technow et al. 2012, 2014; Kadam et al. 2016, 2021; Larièpe et al. 2017; Kadam and Lorenz 2019; Seye et al. 2020; González-Diéguez et al. 2021) and has expanded across diverse mating systems (self- and cross-pollinated crops and clonally propagated species), including wheat (Lado et al. 2017; Yao et al. 2018; Trini et al. 2020; Dreisigacker et al. 2024), rice (Labroo et al. 2021a), barley (Philipp et al. 2016; Osthushenrich et al. 2018), cassava (Wolfe et al. 2021), potato (Endelman 2024), strawberry (Whitaker et al. 2012; Zingaretti et al. 2022; Sleper et al. 2025), and others.

Several lessons from other crops translate directly to sugarcane. General GS model principles (GBLUP effectiveness for polygenic traits) are expected to hold (Meuwissen et al. 2001; Crossa et al. 2017). Training-population design principles (relatedness, cross-validation) are also directly applicable (Hickey et al. 2014; Fritsche-Neto et al. 2018), as is optimal-contribution selection (OCS) theory for balancing gain and diversity (Meuwissen 1997). In addition, multi-stage integration frameworks developed in cassava and potato for clonally propagated crops provide templates for embedding cross prediction within selection pipelines (Werner et al. 2023).

However, other aspects require modification before application in sugarcane. Maize and wheat cross-prediction methods assume diploid or simple polyploid genetics and known ploidy; these need adaptation for variable polyploidy and aneuploidy (Technow et al. 2012; Kadam and Lorenz 2019; Dreisigacker et al. 2024). Likewise, the within-cross variance prediction framework (UC criterion) has primarily been validated in diploid/tetraploid crops and may underperform in higher-ploidy sugarcane where phasing is uncertain (Allier et al. 2019; Wolfe et al. 2021).

Finally, some cross-species lessons may be misleading if transferred uncritically. Maize GCA/SCA decomposition assumes clear heterotic groups with distinct allele frequency spectra, whereas sugarcane lacks defined heterotic groups and the population structure is more continuous (Mendes de Paula et al. 2020; Labroo et al. 2021b; Zhou 2023). Animal breeding OCS typically assumes very large reference populations with deep, near error-free pedigrees and known ploidy (Meuwissen 1997; Meuwissen and Sonesson 1998; Wang et al. 2017), whereas in sugarcane reference populations are smaller and pedigrees—although traceable for multiple generations and demonstrably useful for parent selection (Atkin et al. 2009)—reflect a narrow founder base and complex interspecific ancestry, and ploidy and dosage are variable (Hoarau et al. 2022). Across crops, predictive performance depends on training data structure (Fritsche-Neto et al. 2018; Melchinger et al. 2023), validation design, the proportion of non-additive variance (Werner et al. 2023), and the presence or absence of clear heterotic groups (Mendes de Paula et al. 2020; Labroo et al. 2021b; Zhou 2023).

## Future progress in sugarcane quantitative breeding

The literature and operational constraints converge on a set of research priorities that are both methodologically substantive and immediately relevant to breeding decisions. These priorities reflect the central challenge of adapting quantitative genetics and genomic tools to a highly polyploid, clonally propagated breeding system.

### Rebuilding family-trial genetics around the observation unit

Family plots in PATs should be modelled as means of sampled full sibs rather than replicated observations of a single genotype. Pedigree or genomic integration should scale Mendelian sampling variance appropriately for plot means. This change clarifies the information content of early-stage data and improves estimation of parental breeding values and cross means.

### Define environments to support stability and decision relevance

Environment definition and G×E modelling in sugarcane should follow the principles outlined in Sect. "Trial structure and statistical challenges across breeding stages", treating environments at a resolution that distinguishes region and year. In practice, this requires deliberate alignment of MET design, environment resolution, and covariance structure, rather than simply substituting an FA model into existing analyses. Within such frameworks, choosing an appropriate FA order becomes critical for capturing both scale and cross-over interactions (Smith et al. 2021b, 2025), and the objective is not only improved overall prediction but explicit estimation of stability so that breeders can identify families and parents that perform reliably under variable climates. Collection of environmental covariates could further support interpretation of stability and performance across environments (Tolhurst et al. 2022).

### Modelling competition effects in multi-environment trials

Interplot competition should be treated as a genetic phenomenon and as residual spatial dependence. Extending IGE and competition modelling from single trials to MET settings is likely to yield meaningful improvements for yield traits in single-row PATs and CATs (Belaber et al. 2021). This requires careful model specification to separate direct genetic effects, neighbour effects, and spatial components, and to assess whether modelling competition changes rankings and prediction accuracy in forward selection.

### Stage-integrated analyses across family and clonal trials, with pedigree and genomics

A major opportunity lies in integrating PAT, CAT, and FAT records within a stage-integrated mixed-model system that supports two complementary breeding objectives: (i) evaluation of genetic merit for parent selection and product development, and (ii) prediction of cross-usefulness through estimates of cross means and within-cross genetic variance. No single breeding stage provides all the information required for either objective. PATs capture family-level means but provide limited information on within-family segregation, whereas FATs contain data from a selected subset of elite clones and therefore do not represent the broader breeding population. By combining PAT, CAT, and FAT data through pedigree-genomic relationships and shared standard genotypes, each stage contributes information unavailable in the others. Computationally, such analyses may be implemented as single-stage mixed models or approximated using the correctly weighted two-stage procedures described in Sect. "Single-stage and two-stage analyses: weighting, covariance, and double shrinkage".

The evaluation analysis integrates CAT and FAT records to estimate individual genetic merit while accounting for the effects of prior selection (Thompson 1973; Smith et al. 2021a). The cross-usefulness analysis combines PAT and CAT data to estimate both cross means and within-cross genetic variance, providing a usefulness criterion that reflects expected performance and the potential to generate superior progeny (Allier et al. 2019; Wolfe et al. 2021). Key challenges include linking different genetic units (family means versus individual clones), accounting for the selection bias inherent in sequential stages, and accommodating differences in trial designs and environments (Wei et al. 2022; SRA 2025a). These linked analyses also provide a natural platform for single-step pedigree-genomic integration (Legarra et al. 2014; Hayes et al. 2021).

Conceptually, these approaches extend standard MET mixed-model approaches rather than requiring a new class of models. PATs, CATs, and FATs are modelled according to their respective designs, with pedigree and genomic relationship providing a common genetic scale across stages and years (Smith et al. 2025). This framework allows uncertainty from early-stage evaluations to propagate naturally through subsequent analyses and selection decisions. Traits can be analysed independently and combined through established selection indices (Thompson and Meyer 1986; Jackson et al. 2022; Wei et al. 2022), while genomic regions of known biological importance can be incorporated as additional information where justified (Shi et al. 2019; Akohoue et al. 2026).

**Fig. 3:**
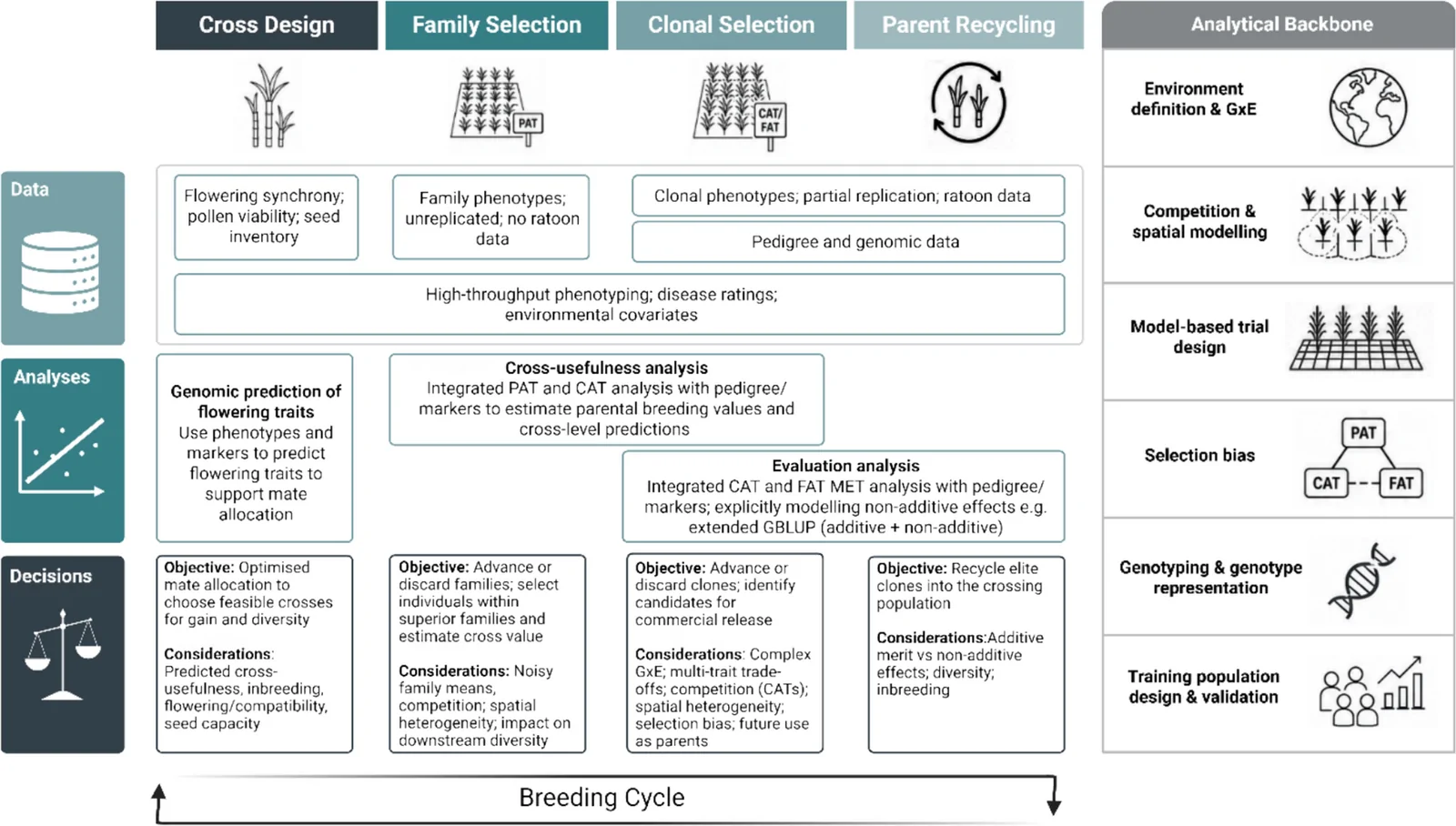
A prospective systems-based framework for mate optimisation in sugarcane breeding. This decision-centred framework links cross design, family selection in progeny assessment trials (PATs), clonal selection in clonal assessment trials (CATs) and final assessment trials (FATs), and parent recycling to genomic prediction and optimised mate allocation in the Australian sugarcane breeding programme. Rows summarise key data inputs, analytical steps, and resulting breeding decisions. Key analytical steps include genomic prediction of flowering traits to support mate allocation (Hayes et al. 2021), cross-usefulness analysis integrating PAT and CAT data to estimate parental breeding values and cross-level predictions, and evaluation analysis integrating CAT and FAT multi-environment trial data for commercial evaluation and parent recycling. The right-hand panel highlights cross-cutting analytical backbone components—environment definition and genotype-by-environment interactions (G×E), competition and spatial modelling, model-based trial design, selection bias, genotyping and genotype representation, and training-population design and validation—that underpin accurate prediction and robust decisions across the breeding cycle. Created in BioRender. Rigby, A. J. (2026) https://BioRender.com/x1elgno

In practical terms, stage-integrated analyses aim to reduce information loss between breeding stages, strengthen connectivity across environments and years, and provide more coherent inputs for parent recycling and genomic mate allocation than traditional stage-wise analyses (Smith et al. 2021a). Figure 3 illustrates a prospective systems-based decision framework linking selection stages, genomic prediction and mate optimisation via shared data streams and an analytical backbone for G×E, competition, trial design, genotyping, and training-population design.

### Cross prediction under constraints: from concept to operational decision support

With improved models for family and clonal stages alongside more accurate relationship matrices, the prediction of untested crosses becomes a realistic next step. Cross prediction should prioritise outputs that map directly onto operational decisions. These include expected family means, the statistical uncertainty of those predictions, and penalties for relatedness that proxy inbreeding depression (or capture potential benefit of genome-wide heterozygosity) (Aliloo et al. 2017; Yadav et al. 2021b). Feasibility constraints as defined above (Fig. 3) should be embedded into the prediction framework so that recommended crosses are both biologically and logistically executable.

### Training-population design tailored to sugarcane realities

Evidence from cross-prediction studies in other crops indicates that predictive accuracy depends on the number of parents, the number of crosses per parent, trait heritability, and the proportion of non-additive variance (Melchinger et al. 2023). Furthermore, model-based trial designs that account for parental representation and relatedness are expected to improve the efficiency and predictive value of the training population (Cullis et al. 2020). Sugarcane constraints make deliberate design more important. Strategies include maximising parental representation while retaining sufficient cross replication, prioritising genotyping that links family and clonal stages, and evaluating models under appropriate prediction scenarios that reflect decision timing (Stringer et al. 2011a; Cullis et al. 2020). While approaches such as sparse METs or optimised trial design (Cullis et al. 2020) may resolve some limitations, their implementation in sugarcane must respect regional relevance and biosecurity regulations: crosses selected for a specific production region should be tested within that region, as material cannot be freely shared across biosecurity borders (Business Queensland 2025).

Taken together, these priorities support a shift from stage-by-stage analyses and late-stage GS towards integrated, design-consistent models that inform early family selection, parent recycling, product development, and cross design.

### What breeders can implement now vs what remains a research priority

The foregoing sections converge on a set of recommendations that differ in their technical readiness and the evidence base supporting them. Table 2 summarises these into two categories: steps that can be implemented with current tools and evidence, and longer-term research priorities that require further development, validation, or infrastructure investment before operational deployment.

**Table 2:** Genomic and quantitative genetic methods and technology for sugarcane breeding

Status	Relevant breeding decision point(s)	Methodological framework
What breeders can implement now	Cross design and parent recycling	GEBV-based parent ranking (GCA) for cross mean prediction to reduce reliance on proven crosses; tools available such as *SimpleMating* and *AlphaMate*
	Family selection, clonal selection, and parent recycling	Use genetic relatedness to inform the allocation of genotypes to plots at the design stage, ensuring the trial layout is optimised for the mixed model that will be used in analysis Cullis et al. (2020)
		Define environments at region × year × crop-cycle resolution rather than region alone; immediate improvement to G×E modelling quality with existing data
		Fully efficient single-stage MET analysis of plot-level data across environments (avoids information loss and double shrinkage; feasible with current mixed-model software and computing) Gogel et al. (2018)
		Modelling competition (IGE) in single-trial PAT/CAT analyses to improve TCH estimates; established methodology Stringer et al. (2011b)
	Clonal selection and parent recycling	Continuous-genotype encoding (normalised array intensities) as a low-cost improvement to genomic prediction for CCS and fibre traits to support selection for commercial release or parent recycling Yadav et al. (2024)
		ssGBLUP for genotyped elite clones to support selection for commercial release or parent recycling; modest but consistent improvements over pedigree-only or genomic-only models at the FAT stage Hayes et al. (2021)
	Cross design, family selection, clonal selection, and parent recycling	Apply selection index for multi-trait ranking from independent single-trait EBVs; compatible with existing programmes and avoids high-dimensional multivariate model complexity
Longer-term research priorities (further validation/development needed)	Cross design and parent recycling	Genome-informed mate allocation incorporating phased haplotypes and prediction of both cross means and within-cross variances; contingent on reliable high-ploidy haplotype phasing at ~12× dosage
		Flowering-trait genomic prediction at scale to guide photoperiod scheduling; current evidence limited to a single study Hayes et al. (2021)
		Systematic prospective evaluation of cross-prediction accuracy in operational sugarcane crossing programmes; currently absent from the published literature
		Dosage-aware prediction of within-cross variance and implementation of polyploid-specific inbreeding control at operational scale
	Family selection, clonal selection, and parent recycling	Competition modelling extended to full MET frameworks in PATs and CATs; conceptually motivated but not yet implemented in sugarcane multi-environment settings
		Single-stage MET mixed-model analysis integrating family and clonal stages (PAT, CAT, FAT) to remove stage-wise selection bias; conceptually achievable, but currently limited by different scales and designs between family and clonal data, and would require substantial method development, infrastructure, and software integration
	Cross design, family selection, clonal selection, and parent recycling	Aneuploidy-aware genotype calling and GRM construction; requires development of calling tools validated at variable local ploidy in sugarcane
		Biologically informed models using R570-anchored QTL/GWAS regions; enabled by the polyploid assembly but not yet validated for GS in operational pipelines

Tools and technology are grouped into those that can be implemented immediately in operational programmes and those that remain longer-term research priorities requiring further development and validation and are mapped to their relevant breeding decision points and pipeline stages

## Conclusions

Sugarcane improvement faces a distinctive combination of constraints: a highly polyploid and aneuploid genome, substantial non-additive genetic variance, long breeding cycles, and early-stage phenotypes that are measured as family plot means rather than individuals. These realities limit the effectiveness of conventional selection and constrain straightforward transfer of genomic prediction and cross-prediction methods developed for diploid, inbred hybrid systems.

Nonetheless, recent advances indicate a clear pathway to faster and more reliable genetic gain. The most immediate gains are likely to come not from adding new model classes in isolation, but from aligning statistical models with the biological observation unit, improving multi-environment definitions, modelling competition explicitly, and moving from stage-wise to stage-integrated mixed-model analyses that incorporate pedigree and genomics fitted within a fully efficient single-stage MET mixed-model framework (Sect. "Single-stage and two-stage analyses: weighting, covariance, and double shrinkage"). On this foundation, genomic prediction of cross performance and constraint-aware mate allocation can reduce reliance on proven crosses, improve exploration of new combinations, and manage inbreeding depression while respecting the operational constraints of flowering and compatibility.

Overall, the literature supports a transition from fragmented, stage-wise analytical pipelines and late-stage genomic selection towards integrated, biology-consistent breeding frameworks. By linking family evaluation, clonal testing, genomic prediction, and cross design within a unified analytical system, these approaches provide a more coherent basis for early selection, parent recycling, and strategic crossing design. Such integration is not merely a statistical refinement but a fundamental shift in breeding strategy, with the potential to accelerate genetic gain while improving the efficiency, robustness, and long-term sustainability of sugarcane improvement programmes.

## Data Availability

No datasets were generated or analysed during the current study.
